# Longitudinal trends in blood concentrations of clozapine and norclozapine, inflammatory markers, and clinical outcomes in Japanese patients: a 12-week prospective study

**DOI:** 10.3389/fpsyt.2026.1806564

**Published:** 2026-03-11

**Authors:** Masaru Nakamura, Takahiko Nagamine, Honami Yokoe, Yusuke Shimomura

**Affiliations:** 1 Department of Psychiatric Internal Medicine, Cocoro Hospital Kusatsu, Hiroshima, Japan; 2 Department of Psychiatric Internal Medicine, Sunlight Brain Research Center, Yamaguchi, Japan; 3 Department of Pharmacy, Cocoro Hospital Kusatsu, Hiroshima, Japan

**Keywords:** clozapine, interleukin-6, Japan, metabolic syndrome, norclozapine, therapeutic drug monitoring (TDM), treatment-resistant schizophrenia

## Abstract

**Introduction:**

Clozapine (CLZ) is the gold standard for treatment-resistant schizophrenia (TRS), but its use in Japan is limited by strict monitoring and titration-phase adverse events. This prospective 12-week study evaluated longitudinal trends in CLZ/norclozapine (NCLZ) levels, inflammatory markers, metabolic indices, and psychiatric symptoms.

**Methods:**

Twenty-one inpatients with TRS were analyzed. To focus on standard titration, patients with inflammatory symptoms (e.g., fever) requiring discontinuation were excluded. Serum CLZ and NCLZ were measured weekly via LC-MS/MS. Clinicians were blinded to these levels; dosing was guided solely by clinical observation. IL-6, HOMA-IR, TG/HDL-C ratios, and PANSS scores were assessed at baseline and designated intervals.

**Results:**

CLZ and NCLZ concentrations increased throughout the 12-week period. Significant sex differences emerged in CLZ concentration-to-dose (C/D) ratios, with females exhibiting significantly higher levels than males starting at week 2 (p < 0.05). While positive symptoms significantly improved (p < 0.05), no specific longitudinal correlations were found between CLZ/NCLZ levels and changes in IL-6, metabolic indices, or total PANSS scores.

**Discussion:**

A “start low, go slow” titration approach can effectively achieve therapeutic concentrations even without real-time therapeutic drug monitoring. However, the significantly higher concentrations observed in female subjects suggest that more cautious dose titration is necessary for female patients, likely due to hormonal influences on metabolic enzymes. Further research is needed on the relationship between clozapine dosage, plasma concentrations and inflammatory side effects.

## Introduction

Clozapine (CLZ) stands as the gold standard for treatment-resistant schizophrenia (TRS). Since its introduction to the Japanese market in 2009, it has been the only pharmacotherapy indicated for patients who fail to respond to at least two different antipsychotics (1). Despite its superior efficacy, clozapine remains underutilized in Japan compared to Western nations (2). This discrepancy is largely attributed to the rigorous safety protocols mandated by the Clozaril Patient Monitoring Service (CPMS), which requires mandatory weekly blood draws for the first 26 weeks and an initial 18-week inpatient stay. These regulations are designed to mitigate the risk of life-threatening adverse events, including agranulocytosis, myocarditis, and severe glucose intolerance.

The pharmacological landscape of clozapine is uniquely complex. It is primarily metabolized in the liver by the cytochrome P450 (CYP) 1A2 enzyme, with secondary pathways involving CYP2C19, CYP3A4, and CYP2D6 (3). The primary metabolite, N-desmethylclozapine (norclozapine, NCLZ), is not merely an inactive byproduct but a pharmacologically active compound with potent 5-HT2C antagonist and M1 receptor agonist properties (4). Research suggests that the relationship between parent drug and metabolite—the CLZ/NCLZ ratio—may hold the key to understanding both the cognitive benefits and the metabolic burdens of the treatment (5).

Caucasians with average clozapine metabolism may need from 350 to 600 mg/day to reach the therapeutic range (350 ng/ml) (6, 7). By contrast, in Asian patients with average metabolism, the dose needed for clinical response may range between 150 mg/day for female non-smokers to 300 mg/day for male smokers (8). This “ethnic sensitivity” is hypothesized to stem from a combination of lower body weight, potential genetic polymorphisms in CYP1A2 activity, and environmental factors such as dietary habits. In 2022, the Japanese health insurance system began covering clozapine Therapeutic Drug Monitoring (TDM), yet many clinicians still rely solely on clinical observation.

Furthermore, the early phase of clozapine initiation is often marked by transient inflammatory responses (9). Clozapine-induced fever and eosinophilia occur frequently, often causing clinicians to prematurely discontinue the drug (10). There is a critical need to understand whether these inflammatory surges are dose-dependent or idiosyncratic. While our previous retrospective studies documented these phenomena, they lacked the longitudinal pharmacokinetic data required to establish causality (11, 12). This prospective study was initiated to fill that gap, examining the trends between CLZ/NCLZ levels, interleukin-6 (IL-6), metabolic indices (HOMA-IR, TG/HDL-C), and psychiatric improvement during a 12-week titration period.

## Method

### Study participants and setting

We conducted a 12-week prospective cohort study at COCORO HOSPITAL KUSATSU in Hiroshima, Japan. Subjects were recruited from inpatients diagnosed with TRS according to the CPMS criteria. TRS was defined as an inadequate response to at least two different antipsychotics at sufficient doses for four weeks or an inability to tolerate standard doses. Exclusion criteria included patients unable to provide informed consent and those with pre-existing medical conditions that could confound inflammatory markers, such as active infections, bronchial asthma, or atopic dermatitis.

**Figure 1:**
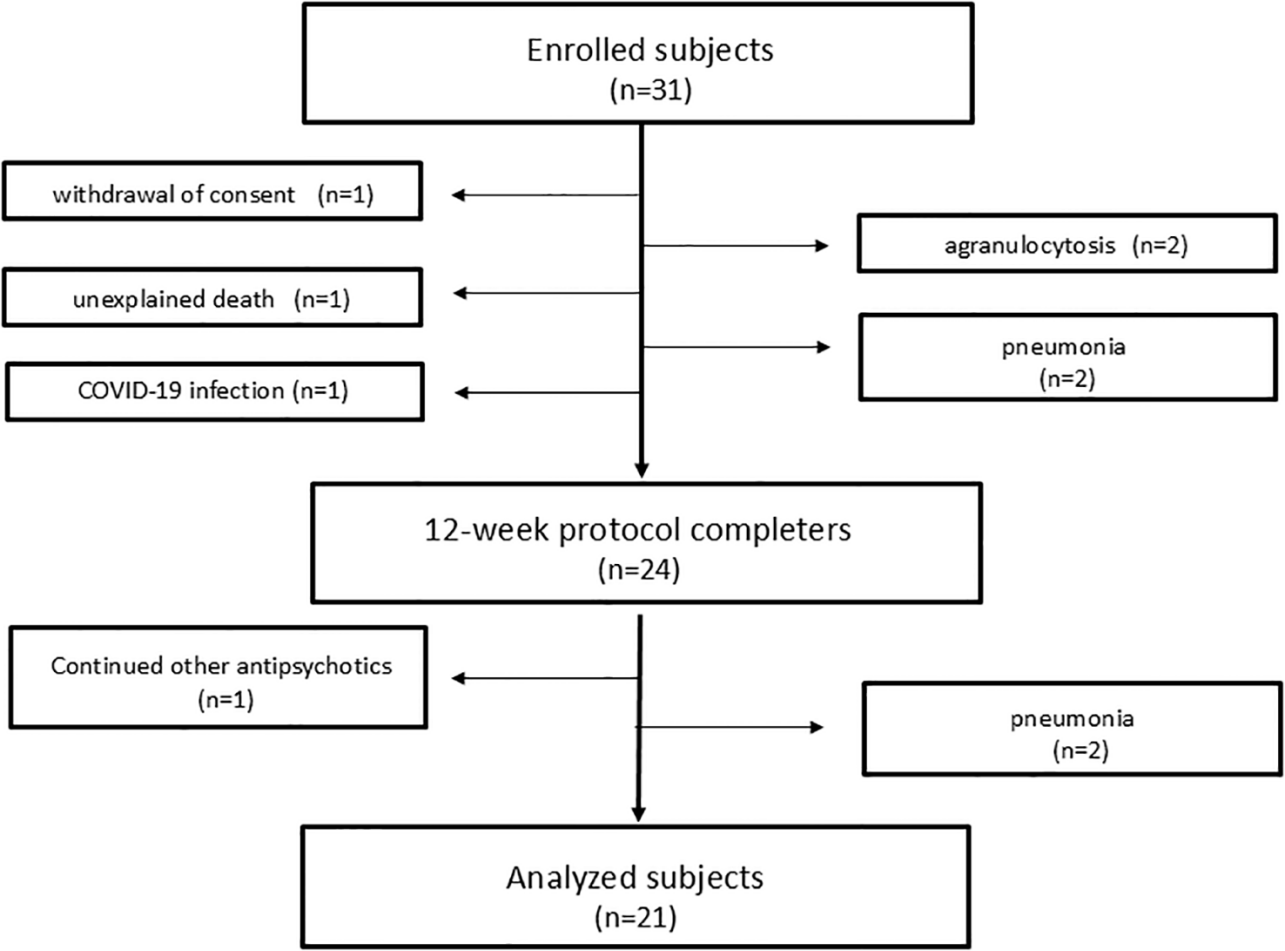
Flowchart of the study design.

As this study aimed to observe longitudinal trends in patients completing the titration protocol, we excluded 10 subjects from the primary analysis (**Supplementary Table 1**). These dropouts were due to agranulocytosis, pneumonia, COVID-19 infection, unexplained death, combination antipsychotic therapy, and withdrawal of consent. This left 21 subjects who did not exhibit inflammatory symptoms necessitating discontinuation for the final analysis (**Figure 1**).

### Drug administration and titration

Clozapine titration followed a “start low, go slow” protocol. Treatment was typically initiated at 12.5 mg, then increased or maintained by 25 mg weekly, reaching a maintenance range of 75–200 mg/day by week 8. Doses were administered one to three times daily. Crucially, clinicians were unable to utilize blood concentrations during clozapine treatment, and measurements were taken later. Dosing decisions were made based on clinical symptoms and mandatory safety monitoring without the aid of real-time TDM.

### Outcome measures and laboratory analysis

Fasting blood samples were collected weekly, exactly 10 to 12 hours after the last dose, to ensure accurate trough level measurements.

1. **Pharmacokinetics:** Serum CLZ and NCLZ concentrations (ng/mL) were measured using liquid chromatography-tandem mass spectrometry (LC-MS/MS) at Fukuyama University. We calculated the CLZ/D ratio, NCLZ/D ratio, and the CLZ/NCLZ ratio.
2. **Inflammatory Markers:** Interleukin-6 (IL-6) levels were determined via ELISA at baseline and at weeks 2, 4, 8, and 12. White blood cell counts, including neutrophils and eosinophils, were monitored weekly.
3. **Metabolic Indices:** Fasting insulin and glucose were measured to calculate the Homeostatic Model Assessment for Insulin Resistance (HOMA-IR). The triglyceride-to-high-density lipoprotein cholesterol (TG/HDL-C) ratio was also monitored.
4. **Psychiatric Symptoms:** The Positive and Negative Syndrome Scale (PANSS) was administered at baseline and week 8 by trained clinicians.

### Statistical methods

A normality test (e.g., Shapiro-Wilk test: p > 0.05) indicated that the data did not follow a normal distribution. For two-group comparisons, the Mann-Whitney U test was applied. For longitudinal changes, the Bonferroni method was applied for multiple comparisons. Differences between pre- and post-treatment were analyzed using the Wilcoxon signed-rank test. Spearman’s rank correlation coefficients were utilized to determine the relationships between dose and blood concentrations. Statistical analyses were performed using EZR software (13). Statistical significance was defined as p < 0.05.

## Results

### Baseline characteristics

The final cohort consisted of 9 males and 12 females. There were no statistically significant gender differences at baseline regarding age (mean age approx. 45), duration of illness, or baseline inflammatory markers, and concomitant use of psychiatric and medical medications (**Supplementary Tables S2**, **S3**).

### Longitudinal pharmacokinetics and sex differences

**Figure 2:**
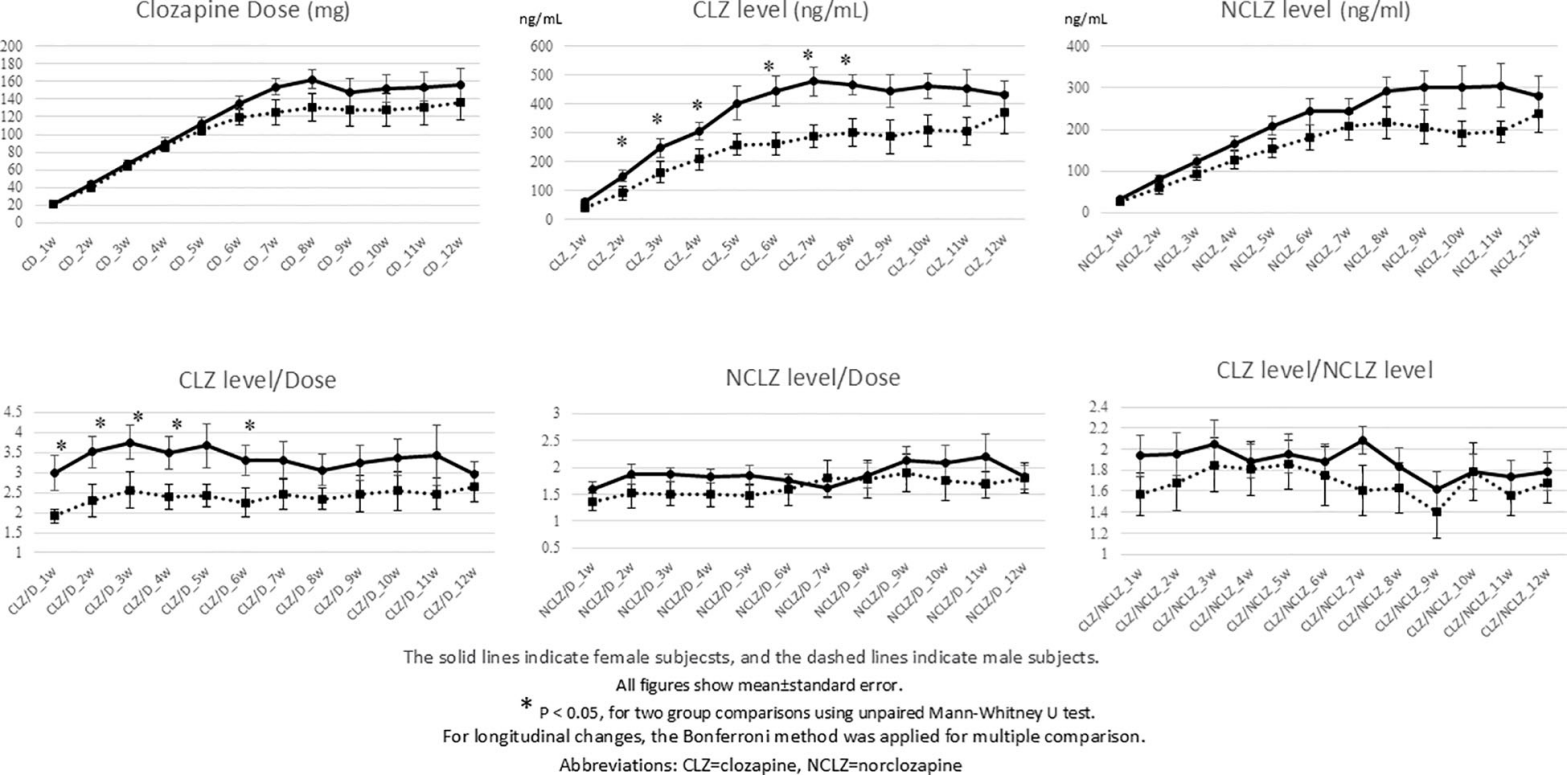
Time-dependent changes in CLZ dose, CLZ level, NCLZ level, CLZ level/Doze, NCLZ level/Dose, and CLZ level/NCLZ level.

By week 12, the mean clozapine dose was approximately 150 mg/day. Notably, even under blinded conditions where clinicians adjusted doses based on clinical response alone, female patients exhibited significantly higher CLZ levels and CLZ/D ratios than male patients starting from week 2 and persisting through week 8 (p < 0.05). This suggests that female patients achieve higher systemic exposure at equivalent doses. The NCLZ/D ratio and the CLZ/NCLZ ratio did not show significant sex-based variance (**Figure 2**).

### Trends in dose and concentration

The relationship between dose and serum levels was recorded over time. While the correlation between these variables appeared to strengthen by week 12 (r = 0.81 for CLZ; r = 0.73 for NCLZ), this study focuses on the descriptive longitudinal trends of these values (**Table 1**).

**Table 1:** Correlations between CLZ dose and blood levels of CLZ and NCLZ.

		CLZ			NCLZ	
	*r*		*p*	*r*		*p*
Week 1	0.567	*	0.008	0.400	NS	0.072
Week 2	0.534	*	0.013	0.500	*	0.021
Week 3	0.341	NS	0.130	0.383	NS	0.087
Week 4	0.263	NS	0.198	0.467	*	0.033
Week 5	0.472	*	0.031	0.591	*	0.005
Week 6	0.455	*	0.031	0.507	*	0.019
Week 7	0.429	NS	0.059	0.434	*	0.006
Week 8	0.628	*	0.002	0.373	*	0.010
Week 9	0.615	*	0.004	0.525	*	0.018
Week 10	0.525	*	0.015	0.408	NS	0.067
Week 11	0.544	*	0.011	0.444	*	0.044
Week 12	0.817	*	<0.001	0.731	*	<0.001

Correlations are expressed with Spearman’s rank correlation coefficients *(r*). *r*>0 a positive correlation.

NS, not significant, *p* value < 0.05 is considered significant and marked with an asterisk.

CLZ, clozapine; NCLZ, norclozapine.

### Inflammatory and metabolic trends

**Figure 3:**
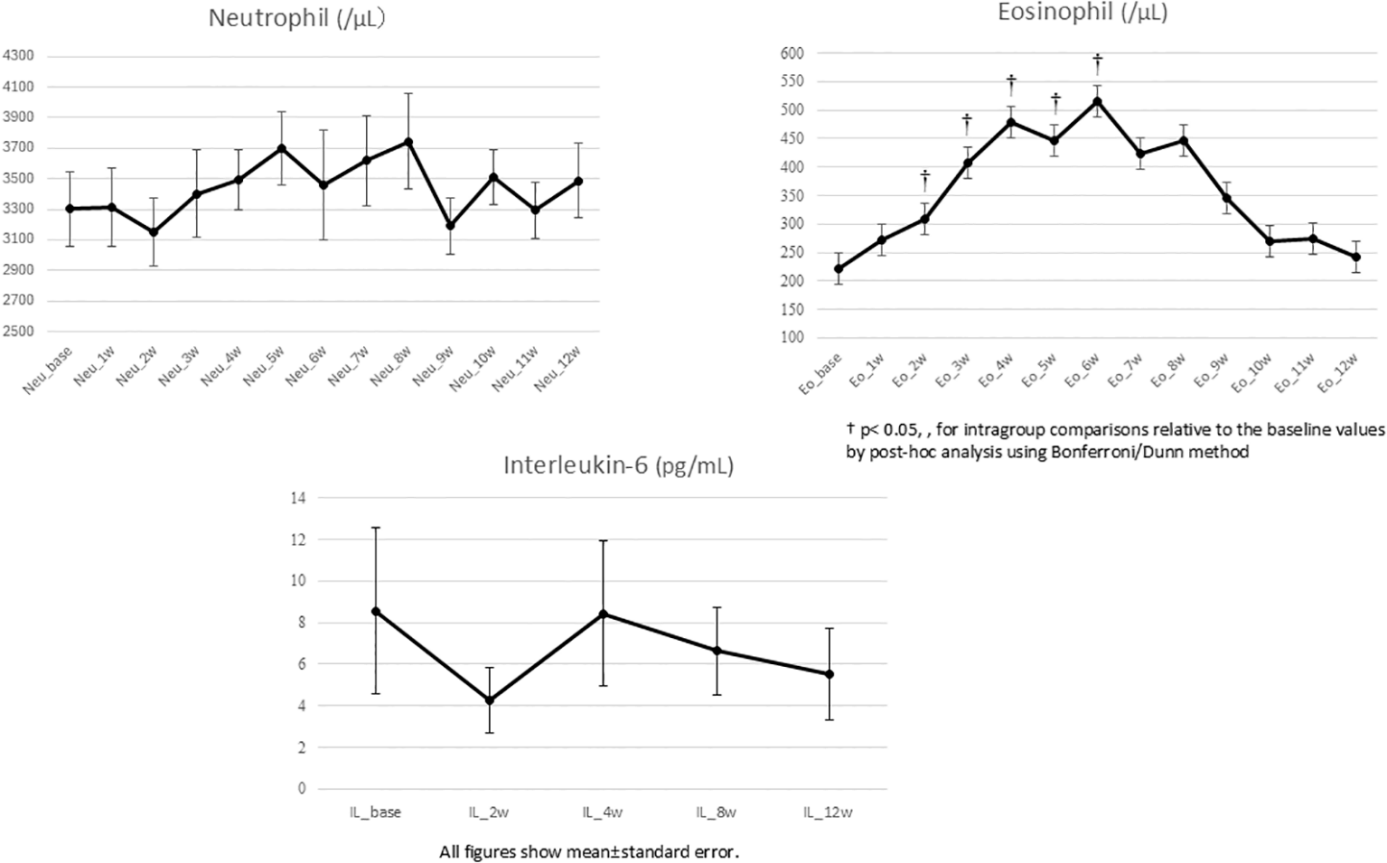
Changes in neurtrophil, eosinophil and interleukin-6.

Neutrophil counts exhibited fluctuation, and no consistent trend was observed. Eosinophil counts showed a significant but transient increase between weeks 2 and 6 (p < 0.05), subsequently returning to baseline. IL-6 levels were highly variable in the first month but trended downward by week 12 (**Figure 3**). Weight and BMI showed an upward trend in week 8, but the increase was not statistically significant. Metabolic indices (HOMA-IR and TG/HDL-C) showed a consistent upward trend throughout the 12 weeks; however, it is unclear whether these differences were statistically significant within the sample size, and no specific correlation between these trends and blood concentrations was identified. (**Supplementary Figure 1**).

### Psychiatric outcomes

PANSS positive symptom scores and total scores showed a significant reduction from baseline to week 8 (p < 0.05), confirming the efficacy of the titration protocol despite the lower doses used (**Supplementary Figure 2**).

## Discussion

This prospective study describes the longitudinal trends of clozapine pharmacokinetics and clinical markers during the 12-week initiation phase in Japanese TRS patients. By focusing on patients who did not exhibit inflammatory symptoms, we were able to observe the physiological trends associated with a conservative titration strategy.

### Sex-based differences and hormonal influence

Our data confirmed significant sex differences, with female patients exhibiting higher CLZ/D ratios. This reinforces the necessity for gender-tailored dosing. Estrogen is a known inhibitor of CYP1A2; consequently, pre-menopausal female patients often require lower doses than male patients to reach the same therapeutic target (14, 15). Interestingly, while CLZ levels were higher in female patients, the CLZ/NCLZ ratio did not differ significantly between sexes. This suggests that while the initial metabolism of clozapine is slower in female patients, the secondary metabolism of norclozapine may be proportionally affected, maintaining a consistent metabolite balance across genders.

Furthermore, even under blinded conditions where clinicians could not utilize TDM for dose adjustment, significantly higher blood concentrations were recorded in female patients starting from week 2, even at the same dose. This finding is highly significant and suggests that more careful dose titration is necessary in female patients to avoid over-exposure. Even with a relatively gradual dose escalation, sufficient blood concentrations (mean: 400 ng/mL) were finally achieved, and improvements in PANSS scores were observed by week 8. This supports the safety and efficacy of slow titration in Japanese patients.

### The independence of inflammatory markers

The analysis of patients without inflammatory symptoms showed no longitudinal correlation between blood concentrations and inflammatory markers. Eosinophil counts peaked between weeks 2 and 6 across the cohort regardless of dose. This suggests that inflammatory surges, when they occur, may be idiosyncratic rather than dose-dependent in the cohort that showed no inflammatory symptoms under extremely slow titration conditions (16). Initially, clozapine may stimulate the release of pro-inflammatory cytokines, but as the patient stabilizes, it may exert a paradoxical anti-inflammatory effect (17). Managing patients through this 6-week window of fluctuation is critical for treatment retention.

### Metabolic trends

While HOMA-IR and metabolic ratios showed an upward trend, this study did not identify a direct correlation with blood concentrations. These metabolic changes—such as the trend toward insulin resistance—may occur even at sub-therapeutic concentrations. Clozapine and norclozapine have high affinity for the 5-HT2C and H1 receptors, implicated in appetite and glucose metabolism (18, 19). This suggests that metabolic monitoring is essential from the onset of treatment, as the “metabolic hit” may be a non-concentration-dependent risk.

### Clinical efficacy at low doses

The significant improvement in PANSS scores at an average dose of 150 mg/day challenges the conventional wisdom that high doses are required for TRS. The average serum level of 400 ng/mL reached by our subjects falls within the international therapeutic window, despite the low dose. This confirms that for the Japanese population, the “therapeutic window” can be reached with minimal exposure, potentially reducing the risk of dose-related side effects. However, close clinical monitoring remains the primary strategy for managing Japanese TRS patients.

### Limitations and future directions

This study is limited by its descriptive nature and the small sample size. Future research should focus on multi-center trials that include genetic screening to further refine clozapine dosing in Asian populations.

## Conclusion

Clozapine blood concentrations increase over time, and levels are significantly higher in female patients than in male patients, potentially influenced by hormonal inhibition of metabolic pathways. This study demonstrates that even without the use of real-time TDM, a “start low, go slow” approach is effective in reaching therapeutic targets. However, changes in inflammatory markers may not be predictable based on plasma concentration alone. Further research is needed on the relationship between clozapine dosage, plasma concentrations and inflammatory side effects.

## Data Availability

The original contributions presented in the study are included in the article/**supplementary material**. Further inquiries can be directed to the corresponding author.
