# Supplementary figures and images for: Longitudinal trends in blood concentrations of clozapine and norclozapine, inflammatory markers, and clinical outcomes in Japanese patients: a 12-week prospective study
Source: Front Psychiatry. 2026 Mar 11;17:1806564. doi: 10.3389/fpsyt.2026.1806564 (PMC13014377; doi:10.3389/fpsyt.2026.1806564)

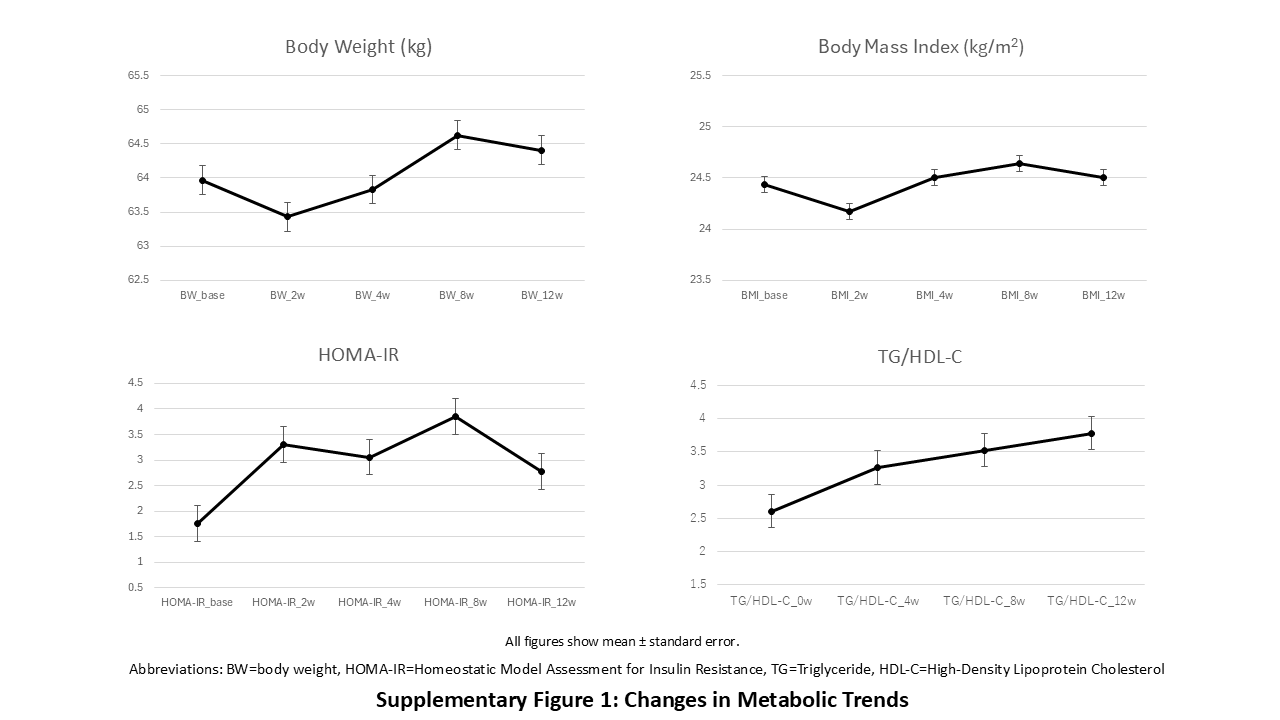

Supplement: Supplementary file 1 [file Image1.tif]

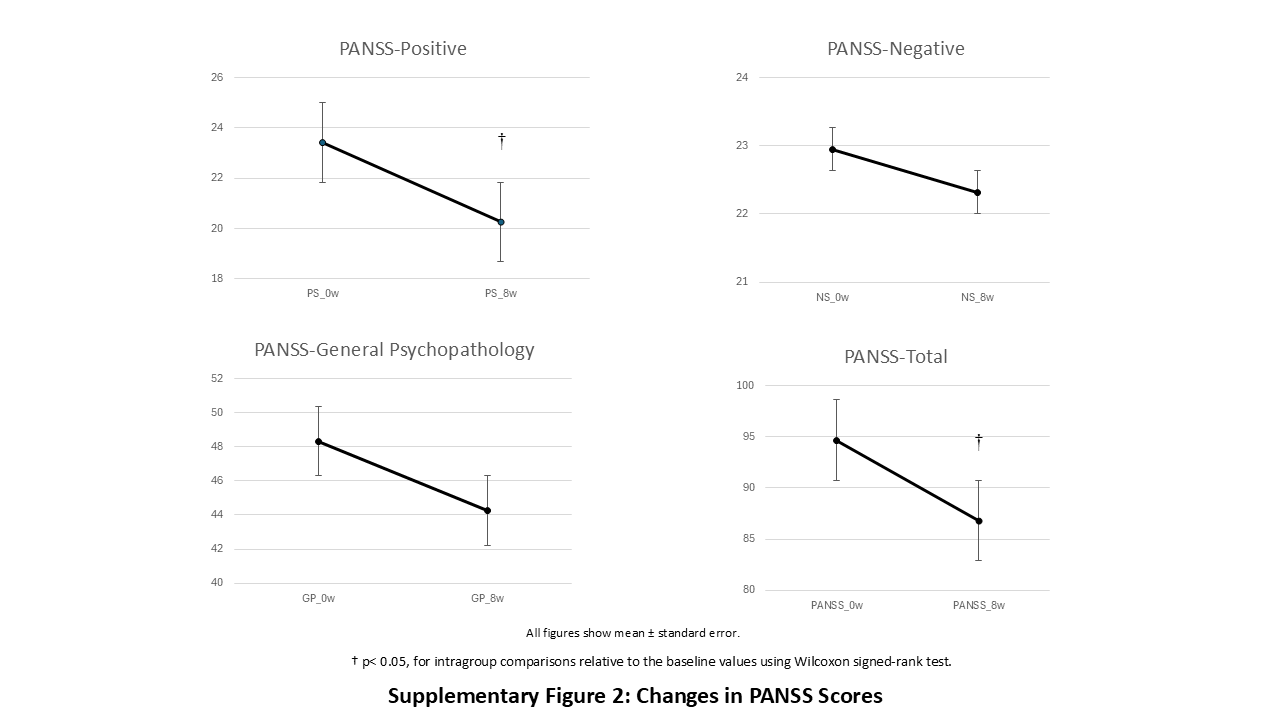

Supplement: Supplementary file 2 [file Image2.tif]
